# Exploring the cardiac ECM during fibrosis: A new era with next-gen proteomics

**DOI:** 10.3389/fmolb.2022.1030226

**Published:** 2022-11-22

**Authors:** Vivek Sarohi, Sanchari Chakraborty, Trayambak Basak

**Affiliations:** ^1^ School of Biosciences and Bioengineering, Indian Institute of Technology (IIT)- Mandi, Himachal Pradesh, India; ^2^ BioX Center, Indian Institute of Technology (IIT)- Mandi, Himachal Pradesh, India

**Keywords:** extracellular matrix, cardiac fibrosis, heart failure, proteomics, mass-spectrometry

## Abstract

Extracellular matrix (ECM) plays a critical role in maintaining elasticity in cardiac tissues. Elasticity is required in the heart for properly pumping blood to the whole body. Dysregulated ECM remodeling causes fibrosis in the cardiac tissues. Cardiac fibrosis leads to stiffness in the heart tissues, resulting in heart failure. During cardiac fibrosis, ECM proteins get excessively deposited in the cardiac tissues. In the ECM, cardiac fibroblast proliferates into myofibroblast upon various kinds of stimulations. Fibroblast activation (myofibroblast) contributes majorly toward cardiac fibrosis. Other than cardiac fibroblasts, cardiomyocytes, epithelial/endothelial cells, and immune system cells can also contribute to cardiac fibrosis. Alteration in the expression of the ECM core and ECM-modifier proteins causes different types of cardiac fibrosis. These different components of ECM culminated into different pathways inducing transdifferentiation of cardiac fibroblast into myofibroblast. In this review, we summarize the role of different ECM components during cardiac fibrosis progression leading to heart failure. Furthermore, we highlight the importance of applying mass-spectrometry-based proteomics to understand the key changes occurring in the ECM during fibrotic progression. Next-gen proteomics studies will broaden the potential to identify key targets to combat cardiac fibrosis in order to achieve precise medicine-development in the future.

## 1 Introduction

Cardiovascular diseases (CVDs) remain the scourge of humanity causing a high number of morbidities and mortalities on a global scale. CVD refers to a broad range of disease manifestations affecting the heart, and subsequently, the vascular system (Murtha et al., 2017). Recent reports from American Heart Association (AHA) indicated ≈17.6 million (95% CI, 17.3–18.1 million) deaths globally were associated with CVD in 2016 accounting for an increase of 14.5% (95% CI, 12.1%–17.1%) deaths within a decade (from 2006) (Benjamin et al., 2019). Heart failure (HF) has remained one of the major contributors to the global burden of CVD. HF refers to the significant decline of the cardiac output (pumping capability of the organ) leading to dysfunction of the organ. According to NHANES data, in between 2009 and 2012, an estimated 5.7 million adults ≥20 years of age had HF and the prevalence increased to an estimated 6.2 million from 2013 to 2016 (Virani et al., 2020). According to the National Heart Failure Registry (India), almost 1.3 million (1% of the population) people get affected by HF annually (ICC NHFR Report, 2022). The prevalence of heart failure has risen over time among elder individuals (Savarese and Lund, 2017). Developed countries have a 1–2% incidence of heart failure but heart failure incidence in people older than 70 years has increased to 10%. It has only a 35% survival rate for patients after their first diagnosis (BLEUMINK et al., 2004). Interestingly, HF patients in India are much younger and have higher morbidity and mortality compared to the Western world (Chaturvedi et al., 2016; Dokainish et al., 2017). Clinical management of HF is quite challenging due to the lack of a precise therapeutic regimen. The cost of heart transplantation is still beyond the reach of the general population. Despite rigorous glycemic control, symptomatic lipid-lowering drugs, ion-channel blockers, and common use of renin–angiotensin–aldosterone system (RAAS) inhibitors, the prevalence of HF progression has remained steady, a fact that emphasizes the need for better therapies to stop HF progression (Domanski et al., 2003; Granger et al., 2003; Klotz et al., 2007). One of the most common pathophysiological characteristics that underpin most CVDs predisposing to heart failure is cardiac fibrosis. Cardiac fibrosis is a condition in which fibrous proteins (collagens, elastin, fibronectin, *etc.*) get excessively deposited in the extracellular matrix (ECM) of heart tissues (Tian et al., 2017). This deposition subsequently leads to stiffness, overgrowth, and scarring in the heart tissues resulting in heart failure (Weber and Brilla, 1991). ECM remodeling resulting in cardiac fibrosis is one of the significant processes involved in the progression of most of the CVDs causing HF. Cardiac fibrosis has emerged as one of the major causes of death due to tissue fibrosis accounting for almost ∼800,000 deaths per year globally (Hinderer and Schenke-Layland, 2019). Cardiac fibrosis is characterized by the development and relentless progression of fibrosis in the myocardium compartments in response to multiple pathogenic stimuli including oxidative stress, hypertension, hypoxia, and inflammation. Assembling the knowledge of intracellular signaling driving the excessive production of ECM components along with the biochemical remodeling in the extracellular space will provide a better understanding of the cardiac fibrosis mechanism. The exact composition of ECM differs in different tissues of different organs (McCabe et al., 2022). Since the ECM composition is varied in different tissues; it is of sheer importance to obtain an in-depth analysis of the myocardium matrisome to understand the pathophysiology of cardiac fibrosis. High-throughput techniques are required to perform in-depth ECM analysis. Next-gen sequencing (NGS) and proteomics are two techniques that are capable of performing in-depth analysis; although NGS-based workflows (mainly RNA-Seq analysis) provide a vivid snapshot of the gene expression during cardiac fibrosis (Faita et al., 2012; McLellan et al., 2020). On the contrary, the expressional abundances, post-translational modifications (hydroxylations, glycosylations, *etc.*), proteolytic processing, protein assembly network (crosslink analysis) of the core, and associated ECM proteins can only be depicted with the mass-spectrometry (MS)-based approaches (Barallobre-Barreiro et al., 2012; Terajima et al., 2014; Basak et al., 2016; Sarohi et al., 2022b). Mass-spectrometry (MS)-based proteomics has been useful in identifying primary sequences, protein–protein interactions, and PTMs (Aebersold and Mann, 2003). Conventional proteomics analysis of ECM matrisomes is filled with several challenges (Chang et al., 2016). A very high abundance of collagens present in ECM increases the dynamic range in detecting low-abundant ECM proteins. Furthermore, the insoluble nature of the ECM matrisome, highly post-translationally modified peptides (of collagens and elastin), and the presence of crosslinked peptides add more challenges to generate MS-amenable peptides (Basak et al., 2016; Chang et al., 2016; Hedtke et al., 2019; McCabe et al., 2022). Recently, modified biochemical methods for ECM proteomics sample preparation, varied fragmentation methods with high-resolution MS, and optimized bioinformatics workflows have made significant advancements to analyze ECM in-depth (Garcia-Puig et al., 2019; Taha and Naba, 2019; McCabe et al., 2021).

In this review, we summarize the role of different ECM components during cardiac fibrosis progression leading to heart failure. Furthermore, we highlight the importance of applying next-gen proteomics (NGP) to understand the key changes occurring in the ECM during fibrotic progression. NGP will provide greater molecular-level details regarding the different activated pathways and biochemical processes during cardiac fibrosis leading to the broadening of the potential to identify key targets to combat cardiac fibrosis.

## 2 Extracellular components altered during cardiac fibrosis

**FIGURE 1:**
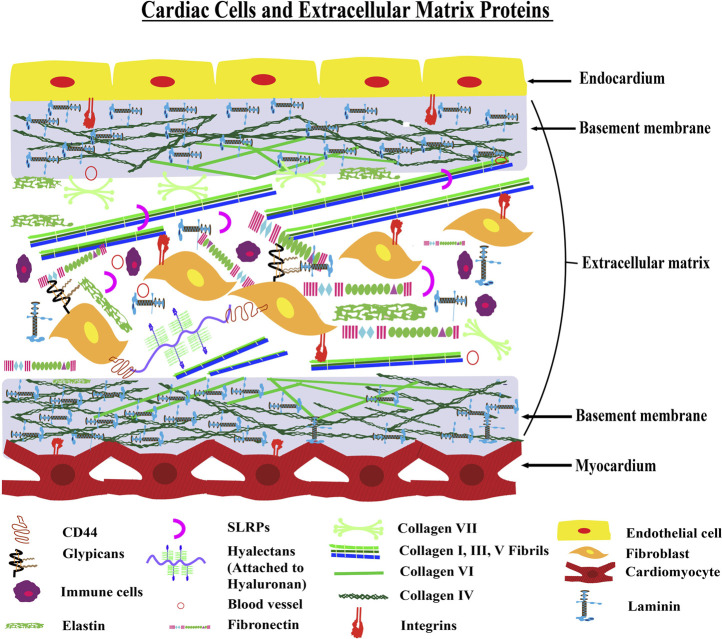
Architecture of Cardiac ECM—The figure shows a model transverse section of the myocardium and endocardium layers of the heart. The myocardium layer contains cardiac muscle cells with a basement membrane and the endocardium layer is made up of endothelial cells with a basement membrane. There is an interstitial matrix between the myocardium and endocardium layers. This interstitial space contains fibroblast and extracellular matrix proteins including collagens, elastin, and fibronectin. Different inflammatory cells are also present in the interstitial matrix conditionally.

The extracellular matrix (ECM) has a tissue-specific structure and function. Cardiac muscle is primarily composed of terminally differentiated tissue-specific cells such as cardiomyocytes, fibroblasts, *etc.* (Figure 1). These cells contribute to the maintenance and deposition of cardiac-specific ECM. The cardiac ECM is a dynamic, robust, and functionally versatile component that forms the non-cellular part of the cardiac muscle. It acts not only as an architectural scaffold that supports the cardiac cells but also actively participates in the development and differentiation of cardiac and vascular cells (Bloksgaard et al., 2018) (Jane-Lise et al., 2000).

### 2.1 Collagens

The primary source for the synthesis and deposition of collagens, one of the important components of the myocardium ECM, is the cardiac fibroblast (Villarreal et al., 2009; Hinderer and Schenke-Layland, 2019). There are 44 types of collagen chains present in the human proteome (Nauroy et al., 2018a). A recent proteomic study has shown that collagens have tissue-specific distinct expressional patterns present in the ECM (McCabe et al., 2022). However, collagen I is the most abundant protein in the ECM of almost every tissue in the human body including the myocardium (McCabe et al., 2022). Another proteomic study was done by Barallobre-Barreiro et al.; they identified 19 collagen chains in the human heart. They found the level of collagens (COL1A1, COL1A2, COL3A1, COL5A1, COL5A2, COL6A1, and COL14A1) to be elevated in ischemic and non-ischemic heart disease patients compared to healthy humans (Barallobre-Barreiro et al., 2021a). Interestingly, single-nucleotide polymorphism in basement membrane collagen (COL4A1 and COL4A2) genes has been found to be associated with a higher risk of coronary artery disease (Steffensen and Rasmussen, 2018). In addition to collagen IV, collagen V was also found to be associated with cardiac diseases (Yokota et al., 2020). Yokota et al. performed a tandem mass tag (TMT)-based quantitative proteomic study of cardiac tissues from infarcted mice hearts and clearly depicted that the collagen (COL1A1, COL1A2, COL3A1, COL5A1, COL5A2, COL5A3, COL6A1, COL6A2, and COL6A3) abundances were increased in the infarcted area. They also found that the deletion of collagen V resulted in a paradoxical increase in the size of post-infarction scars in mice. However, the absence of collagen V causes the scar to be less stiff. This less stiffness in the scar is sensed by mechanosensitive integrins. Then, integrin-signaling promotes transdifferentiation of fibroblast into myofibroblast leading to increased scar size. This indicates that collagen V regulates the scar size during post-MI in mice *via* an integrin-dependent manner (Yokota et al., 2020). Collagen I and III make up almost 95% of the total collagen present in the ECM-maintaining tissue structure (Di Lullo et al., 2002; Gagliano et al., 2009; Fedarko, 2014). They provide structural support by transmitting mechanical forces (contractile) throughout the myocardium to maintain cardiac muscle architecture during a regular cardiac cycle. Collagen I and III also contribute to the visco-elastic properties of the myocardium (Bishop, 1998). The increased accumulation of collagens I–III that occurs in the hypertensive heart and HF has been associated mostly with fibrosis (Weber et al., 1994a). A recent report suggested that even macrophages can contribute to fibrosis by producing excessive collagens, suggesting a secondary source for collagen production in the myocardium (Simões et al., 2020). The myocytes and endothelial cells are lined over basal lamina (basement membrane-BM) primarily composed of Type IV and VI collagens. Collagen-IV, the main component of BM and a highly post-translationally modified protein, plays a major role in the cell–matrix interaction thereby regulating cellular differentiation, migration, proliferation, adhesion, and signaling cascade. The signaling cascade is executed *via* the cell–ECM interaction through cell-surface receptors (integrins) with arginine, glycine, and aspartic acid (RGD) sequences present in the triple-helical domain. Col-IV and Col-VI expressions have been shown to be increased in pressure-overloaded rat myocardium leading to cardiac insufficiency by alterations of cell–ECM interactions (Kostin, 2000)^,^ (Chapman et al., 1990). More importantly, collagen post-translational modifications (PTMs) could also play a significant role in cell–ECM interactions (Myllyharju, 2008; Jürgensen et al., 2011; Pokidysheva et al., 2014; Stawikowski et al., 2014; Sipila et al., 2018; Rappu et al., 2019).

### 2.2 Collagen modifications

Collagen biosynthesis involves heavy post-translational modifications (PTMs). Proteomics studies have shown that these PTMs have a role in the stability, function, and communication of collagen I with other ECM components and cell-surface receptors to regulate cell adhesion, migration, and cardiac regeneration (Pokidysheva et al., 2014; Basak et al., 2016; Merl-Pham et al., 2019; Sarohi et al., 2022b). Injury and scars are non-repairable in the mammalian heart due to a lack of regeneration capability (Cutie and Huang, 2021). Contrasting to human cardiac tissues, zebrafish have the ability to regenerate heart tissue (Chen W. C. W. et al., 2016; Sánchez-Iranzo et al., 2018). In an interesting proteomic study done by Sarohi et al., they detected 36 collagen chains in zebrafish heart. The levels of collagen PTMs were found to be altered during the regeneration process in the zebrafish heart (Sarohi et al., 2022b). This hints toward the role of collagen PTMs in the zebrafish heart-regeneration process. Col-IV is more post-translationally modified compared to Col-I. The two amino acids which are majorly modified during collagen biosynthesis are proline and lysine (Myllyharju, 2005; Salo and Myllyharju, 2021). Proline can be either modified into 3-hydroxyproline (3-HyP) or 4-hydroxyproline (4-HyP). Prolyl 3-hydroxylation is catalyzed by prolyl 3-hydrolases. Prolyl 3-hydroxylase 1 (P3H1), P3H2, P3H3, and P3H4 isoforms are present in mammals (Vranka et al., 2004). On the other hand, prolyl 4-hydroxylation is catalyzed by prolyl 4-hydroxylases. Prolyl 4-hydroxylase subunit alpha-1 (P4HA1), P4HA2, and P4HA3 isoforms are involved in prolyl 4-hydroxylation of collagen chains (Salo and Myllyharju, 2021). While 4-HyP provides stability to the collagen structure (Salo and Myllyharju, 2021), the significance of the site-specific presence of 3-HyP in collagen chains has still remained elusive. Lack of 3-HyP in the collagen can cause abnormality in tissue function, embryonic growth, myopia, *etc.* (Cabral et al., 2007; Homan et al., 2014; Pokidysheva et al., 2014; Hudson et al., 2015). Lysine modifications are of diverse types in collagens. Lysine can be catalyzed to hydroxylysine (HyK) by procollagen-lysine, and 2-oxoglutarate 5-dioxygenases (PLODs) (Salo and Myllyharju, 2021), also known as lysyl hydroxylases (LHs). PLOD1, PLOD2, and PLOD3 isoforms are present in mammals. HyK serves as the primary substrate for the O-glycosylation modifications on collagen chains. Hydroxylation of lysines is essential for the assembly of a functional collagen structure. Lack of the lysyl-hydroxylation ability causes muscular abnormalities (Takaluoma et al., 2007). Hydroxylated lysines can be O-glycosylated to galactosyl-hydroxylysine (G-HyK) and glucosylgalactosyl-hydroxylysine (GG-HyK). O-glycosylation of HyKs is catalyzed by enzymes called procollagen galactosyltransferase 1 (COLGALT1) also known as GLT25D1 and COLGALT2 (GLT25D2) (Hennet, 2019; Salo and Myllyharju, 2021). Prolines and lysines are modified in large numbers in collagens. The role of the lysyl and prolyl-hydroxylation and O-glycosylation (G-HyK and GG-HyK) sites in collagens is poorly understood during fibrosis progression. Proteomics provides broader opportunities to identify these PTMs in a site-specific manner in order to study the roles of these PTMs during cardiac fibrosis (Merl-Pham et al., 2019; Sarohi et al., 2022b). Apart from hydroxylation and O-glycosylation, lysine can also be modified to allysines by oxidation. This reaction is catalyzed by lysyl oxidase enzymes. Lysyl oxidase (LOX) is a copper-dependent enzyme that oxidizes peptidyl lysine to peptidyl *a*-aminoadipic-γ-semialdehyde that begins the cross-linking of collagen and elastin in the extracellular matrices (Smith-Mungo and Kagan, 1998). LOX family is comprised of classical LOX and four other LOX-like enzyme isoforms— LOXL1, LOXL2, LOXL3, and LOXL4 (Molnar et al., 2003). Lu et al. showed that inhibition of LOX reduced myocardial fibrosis and cardiac remodeling whereas transforming growth factor-β (TGF-β) induced LOX activity and augmented cardiac fibrosis and heart failure (Lu et al., 2019). Ohmura et al. experimentally proved that the effect of cardiac hypertrophy agonist angiotensin II in the rat increases the LOXL1 mRNA by almost two-fold promoting enhanced myocardial stiffness (Ohmura et al., 2012). Increased crosslinking of collagens during fibrosis is possibly one of the main mechanistic phenomena involved in the progression of heart failure. Yang et al. showed that LOXL2 is up-regulated in the cardiac interstitium during cardiac fibrosis and found abundant levels in the serum of heart-failure patients (Yang et al., 2016). They have also shown that antibody-mediated inhibition of LOXL2 attenuates cardiac fibrosis and dysfunctions (Yang et al., 2016). LOXL3 has also been found to be associated with spinal development. Loss of LOXL3 can cause perinatal death (Zhang et al., 2015). The roles of LOXL3 and LOXL4 in cardiac fibrosis are not clearly understood to date. Recently, Busnadiego et al. experimentally showed that LOXL4 was involved in ECM deposition in endothelial cells during vascular remodeling induced by TGF-β (Busnadiego et al., 2013). These enzymes of the lysyl-oxidase family have emerged as attractive targets for tissue fibrosis. Recently, Santamaria et al. have shown that the LOX family members are increased post-MI and correlated with the accumulation of matured collagen in the infarct zone (González-Santamaría et al., 2016). Thus, understanding the functional role of these enzymes in ECM remodeling during cardiac fibrosis is of utmost importance. Targeted MS-based approaches (such as multiple reaction monitoring-MRM) can be developed to assess the levels of all these five forms of LOX family enzymes at once (Freue and Borchers, 2012). Developing this kind of assay will help achieve isoform specificity in a disease-specific manner. An increase of LOX family enzymes hints toward increased crosslink in the myocardium leading to more stiffened myocardium. Thus, the levels of these LOX-dependent crosslinks may be evaluated in greater detail in several animal models of cardiac fibrosis progression. Furthermore, circulating crosslink (allysine-mediated crosslinks), the measurement may also serve as a potential surrogate marker to assess the stage and severity of cardiac fibrosis among patients.

### 2.3 Fibronectin

Fibronectins (FN) are insoluble glycoproteins secreted by fibroblasts and are associated with the basal lamina of ECM (Schwarzbauer, 1991). Similar to Col-IV, they have binding domains (arginine, glycine, aspartic acid, or RGD motif) for cell-surface receptors, ECM proteins, and proteoglycans. The expression of FN is influenced by mechanical or local factors such as growth factors. The expressions of FN isoform mRNA EIIIB and EIIIA are preceded by an increased expression of TGF-β (Villarreal and Dillmann, 1992) followed by the deposition of collagens I-III in a fibrotic scar (Contard et al., 1991). Fibronectin, deposited in the tissue scar during heart failure (Oliviéro, 2000) is one of the ECM markers for cardiac fibrosis. Genetic inhibition of the FN in mice regulates the excessive deposition of collagens and transdifferentiation of myofibroblasts in the I/R heart failure mice model (Valiente-Alandi et al., 2018). In hypertrophied cardiomyocytes, deletion of FN improves survival (Konstandin et al., 2013).

### 2.4 Laminin

Laminin is a major structural glycoprotein of the basal lamina of cardiomyocytes. Laminin binds with transmembrane receptors present on the sarcolemma of cardiomyocytes and connects with collagen IV, perlecan, nidogen, and fibronectin (Tryggvason, 1993). Laminin transduces the interaction between the cytoskeleton of cardiomyocytes and the remaining is of the basal lamina of cardiomyocytes. It forms the structural component of the basal membrane and connects with extracellular domains and the cytoskeleton or myofibrils. It has different functional domains that bind collagen IV, nidogen, proteoglycans, and transmembrane receptors (Tryggvason, 1993). Merosin (Laminin 2) is abundant in cardiac-striated muscles. Deficiency of Laminin two results in breakage of cytoskeleton-extracellular matrix linkage leading to muscular dystrophy and dilated cardiomyopathy (Campbell, 1995). Sarcolemma properties in failing hearts may be contributed by an imbalance in the decreased or steady-state level of the laminin α2 chain and increased myocyte size (Lundgren et al., 1988). It is also proven that laminin and not fibronectin, is essential for the survival of adult cardiomyocytes in culture conditions (Lundgren et al., 1988). Furthermore, laminin α4 deficiency results in cardiac dysfunction and cardiac vessel dilation leading to heart failure. Laminin deficiency causes loss of interaction between actin of cardiomyocyte and ECM resulting in apoptosis (Wang et al., 2006). Structurally important laminin is essential for cardiac functioning. Impairment in laminin can contribute to the development of cardiac diseases.

### 2.5 Metalloproteinases

Matrix metalloproteinases (MMPs) are zinc-dependent enzymes catalytically active in the extra-cellular space (Gonzalez-Avila et al., 2019). MMPs are responsible for increasing or inhibiting matrix degradation which is a critical regulator of ECM remodeling during cardiac fibrosis (Frangogiannis, 2017). There are six types of MMPs found in ECM: gelatinases (MMP-2 and 9), collagenases (MMP-1, 8, 13, and 18), stromelysins (MMP-3, 10, and 11), matrilysins (MMP-7), membrane-type MMPs (MMP-14, 15, 16, and 17), and other unspecified MMPs. Collagenase and gelatinase MMPs play a crucial role in collagen cleavage in ECM (Frangogiannis, 2017). Collagenases (MMP-1) cleave collagens in fragments that result in the formation of substrates for other less-specific proteases, such as gelatinases (MMP-2). Less-specific MMPs are responsible for the degradation of type IV collagen and fibronectin (Shapiro, 1998). MMP-1 was found to attenuate cardiac fibrosis in suprarenal aortic-banding mice model (Foronjy et al., 2008). A study done on coronary artery-ligated rat revealed that inhibition of MMP-9 improves cardiac function by increasing autophagic flux and regulating ECM remodeling in post-MI chronic heart failure (Nandi et al., 2020). The level of MMP-14 was found to be elevated in swine models with left ventricle fraction shortening compared to the healthy controls (Plavelil et al., 2020). MMPs are regulated by a class of proteins called tissue inhibitors of metalloproteinases (TIMPs). TIMPs are the regulators of cardiac remodeling and myocardial ECM homeostasis. Four types of TIMPs: TIMP-1, TIMP-2, TIMP-3, and TIMP-4 are found in ECM and play a crucial role in cardiac fibrosis progression (Kandalam et al., 2011)^,^ (Kassiri et al., 2009; Takawale et al., 2017)^,^ (Yarbrough et al., 2014). A study performed on an experimental mice model of angiotensin II and pressure overload-induced cardiac fibrosis showed that TIMP-1 deficiency reduces cardiac fibrosis in mice (Takawale et al., 2017). A disintegrin and metalloproteinase with thrombospondin motifs (ADAMTS) is a metalloproteinase enzyme. ADAMTS has catalytic action on procollagen type I, II, III, and IV propeptides. However, the exact role of ADAMTS in the cardiac function is elusive. Interestingly ADAMTS-deficient mice showed exaggeration in the cardiac hypertrophy induced by pressure overload and angiotensin-II (Ang-II) (Wang et al., 2017). This observation indicates that ADAMTS has a role in cardiac hypertrophy regulation.

### 2.6 Cartilage intermediate layer protein

CILP is a secreted matricellular protein that has gained significant interest in the context of cardiac fibrosis recently. CILP has two isoforms—CILP1 and CILP2. CILP (CILP1) was mainly identified as a protein in the mid-zone of the articular cartilage (Lorenzo et al., 1998a). Lorenzo et al. first identified and named the protein on the basis of its immunolocalization in 1998. Although the precursor protein of CILP is a single polypeptide chain, it gets cleaved upon or before secretion by a furin-like protease into two fragments: N-terminal CILP and C-terminal CILP (Lorenzo et al., 1998b). The expression of CILP mRNA can be induced by TGF-β (Mori et al., 2006) whereas CILP-1 binds with TGF-β and inhibits the TGF-β-mediated pathways (Seki et al., 2005). It also acts as an antagonist of IGF-1 (Johnson et al., 2003). CILP is associated with cartilage structure and cartilaginous diseases such as Luber Disc Disease (LDD) (Seki et al., 2005). Masuda et al. found that mRNA expression of CILP was higher in adult (3–4 years old) porcine compared to young (7–10 days old) porcine (Masuda et al., 2001). Lorenzo et al. showed that CILP-1 gene expression was up-regulated in early-stage osteoarthritis (Lorenzo et al., 2004). In contrast to CILP-1, Bernardo et al. showed that CILP-2 gene expression was down-regulated. Previously, CILP was only considered as a protein involved in cartilage, but in 2012, Barallobre-Barreiro found in a proteomic analysis that CILP-1 is also involved in cardiac ECM remodeling (Barallobre-Barreiro et al., 2012; Van Nieuwenhoven et al., 2017). In 2018, Zhang et al. tried to experimentally establish that CILP-1 helps to reduce cardiac fibrosis through the TGF-β signaling pathway (Zhang et al., 2018). But recently, a study involving TMT-based proteomics showed that CILP-1 promotes cardiac fibrosis after MI interfering through the mTORC-1 pathway (Zhang et al., 2021). Thus, the role of CILP-1 during cardiac fibrosis has been established. Further exploration of the role of CILP-1 in different pathologies related to cardiac fibrosis is required for a better understanding of the function of CILP-1 in cardiac pathologies.

### 2.7 Transglutaminase

Transglutaminases are in the Ca^2+^-dependent enzyme family that involve post-translation modification of proteins where isopeptide crosslinking is formed due to transamidation of glutamine residue (Greenberg et al., 1991). Among the other transglutaminases, tissue transglutaminase-2 (TGM2) is mostly abundant in cardiomyocytes, macrophages, and vascular cells (Sane et al., 2007) and up-regulated in various fibrotic diseases (Benn et al., 2019). Multimerization of latent TGF-β binding proteins (LTBPs) is essential for the proper activation of profibrotic cytokine TGF-β in fibrosis and the process is enhanced by transglutaminase-2 (Troilo et al., 2016). Transglutaminase-2 participates in myofibroblast activation through the TGF-β pathway and plays a critical role in various fibrotic diseases including lung, kidney, and heart diseases. Wang et al. showed that inhibition of transglutaminase-2 attenuates cardiac fibrosis in Ang-II model (Wang et al., 2018).

### 2.8 Proteoglycans associated with cardiac fibrosis

Proteoglycans are densely glycosylated proteins. In the glycosylation process, glycosaminoglycan (GAG) chains are attached to serine residues of proteoglycans. Proteoglycans have distribution in the plasma membrane, extracellular matrix, and intracellular structure of cells (Frevert and Wight, 2006). In the extracellular matrix, proteoglycans have a quite complex distribution. Proteoglycans form complexes with collagen and hyaluronan (Frevert and Wight, 2006). On the basis of size, structure, and localization, proteoglycans are classified into four groups (AD et al., 2010).

#### 2.8.1 Syndecans: cell-surface proteoglycans

Syndecans are proteoglycans present on the surface of cells. The syndecan group has four transmembrane receptors. The extracellular domain of these receptors interacts with growth factors (such as TGF-β, Ang-II, *etc.*) available in ECM (Couchman, 2003). In cardiac ECM, syndecan-1 and syndecan-4 play a significant role in the healing of MI and protection from injury and dysfunction. Syndecan-1 plays a cardioprotective role by enhancing MMP-2 and MMP-9 activities and inhibiting leukocyte migration and trans-endothelial adhesion (Vanhoutte et al., 2007). The cardioprotective role of syndecans-4 has been observed in many experiments. Without syndecans-4, endothelial cell proliferation tube, formation, and fibroblast impairment occur but overexpression of syndecans-4 impairs cardiac function (Matsui et al., 2011). However, in cardiac-remodeling experiments, syndecan-1 and syndecan-4 have been also shown to play other roles. Higher concentrations of extracellular syndecans-1 exaggerate fibrosis by increasing the expression of CCN-2 resulting in enhanced collagen deposition during angiotensin II-induced pressure overload in mice (Schellings et al., 2010). Overexpressed syndecan-4 increases cardiac rupture after MI by disrupting the granulation tissue formation (Matsui et al., 2011).

#### 2.8.2 Hyalectans

Hyalectans are proteoglycans with lectin-binding properties. Hyalectans have the ability to bind with a different kind of GAG with no sulfate called hyaluronan. Versican is a proteoglycan of the hyalectan group (Iozzo and Murdoch, 1996). It is ubiquitously expressed in the human body. Studies on versican function in cardiac tissues showed that it is useful in the cardiac ECM during cardiac development (Cooley et al., 2012). Versican has the ability to bind with many components of ECM and inflammatory factors present in the ECM including fibulin (1 and 2), collagen I, hyaluronan, tenascin, fibronectin, and various chemokines (Wu et al., 2005). Studies on versican showed that versican has an important role in modulating inflammatory responses. This indicates that versican may have some role in the pathophysiology of many cardiac conditions (Kawashima et al., 2000).

#### 2.8.3 Perlecan: basement membrane proteoglycans

Perlecan is a proteoglycan of the basement membrane. It is predominantly found in vascular ECM. Perlecan has both pro-angiogenic and anti-angiogenic properties (Mongiat et al., 2003; Iozzo et al., 2009). Fibroblast growth factor-2 (FGF-2) mediates the functions of perlecan. Perlecan plays a crucial role in cardiac development (Zhou et al., 2004; Sasse et al., 2008). A deficiency of perlecan in mice impairs cardiac development and is also critical for maintaining stability in the adult heart upon injury (Sasse et al., 2008). The role of perlecan in the pathophysiology of cardiac diseases is elusive.

#### 2.8.4 Small leucine-rich proteoglycans

Small leucine-rich proteoglycans (SLRPs) are proteoglycans with small molecular weights (36–42 KDa). SLRPs are rich in leucine. In cardiac ECM, SLRPs have interactions with various cytokines, growth factors, cell-surface receptors, ECM proteins, collagens, TLRs, epidermal growth factor receptors and insulin growth factor receptors, low-density lipoprotein receptors, and TGF-β. The ability of SLRPs to interact with several components indicates the involvement of SLRPs in a wide range of cellular functions and pathophysiologic responses, including inflammation, collagen fibril assembly, fibrosis, cell transdifferentiation, and atherosclerosis (Cabello-Verrugio et al., 2012) (Hildebrand et al., 1994). SLRP decorin works as an antagonist of TGF-β. Expression of the decorin gene in spontaneously hypertensive rats reduces the profibrotic TGF-β expression resulting in a reduction in fibrosis. Individuals having aortic stenosis show increased expression of SLRP osteoglycin and it is strongly correlated with the left ventricular mass (Merline et al., 2009). This correlation signals that SLRPs have some role in cardiac remodeling and SLRPs can be further explored to investigate their exact role in cardiac remodeling.

### 2.9 Glycoproteins associated with cardiac fibrosis

Glycoproteins are proteins glycosylated with oligosaccharides at amino side chains. Glycoproteins are widely present in prokaryotic and eukaryotic cells. In cardiac ECM, glycoproteins play a non-structural role. The following glycoproteins are associated with cardiac remodeling.

#### 2.9.1 Thrombospondins

Thrombospondin (TSP) is a family of five glycoproteins. Thrombospondins have anti-angiogenic properties**.** Thrombospondins are subdivided into two groups. Trimeric TSP-1 and TSP-2 are in the first group and pentameric TSP-3, TSP-4, and TSP-5 are in the second group (Iruela-Arispe et al., 1993). TSPs are only secreted during injury or cardiac development. During cardiac development, TSP-1 is expressed. TSP-2 is expressed in connective tissue whereas TSP-3, TSP-4, and TSP-5 are organ-specific and are expressed in the brain, cartilage, lung, and the nervous system. TSP-1 and 2 play significant roles in cardiac pathology. TSP-1 plays a protective role after MI by inhibiting angiogenesis and facilitating the activation of TGF-β. Deletion of TSP-1 in canine and murine-reperfused infarction models resulted in a prolonged post-infarction inflammatory response and extensive post-infarction remodeling (Frangogiannis et al., 2005). TSP-1 is also a potent angiostatic mediator in the context of the diabetic heart. TSP-1 can mediate vascular rarefaction by enhancing angiopoietin-2 expression in diabetic hearts (Gonzalez-Quesada et al., 2013). TSP-2 is required for maintaining the integrity of the myocardial matrix during a pressure overload condition. It protects the cardiac matrix during pressure overload induced by Ang-II infusion by inhibiting the MMP activity (Schroen et al., 2004). TSP-2 has protective effects on the ageing heart; it activates pro-survival Akt signaling and inhibits MMP activity (Swinnen et al., 2009).

#### 2.9.2 Tenascins

Tenascins are multimeric glycoproteins present in the extracellular matrix. Their repeated units make different structures similar to hepta repeats, epidermal growth factor (EGF)-like repeats, fibronectin type III repeats, and one globular domain which matches with fibronectin. Three types of tenascins are present in humans. Tenascin-R and tenascin-C are present in a large number of developing tissues including the nervous system (Chiquet-Ehrismann, 1995). Tenascin-X mRNA and proteins are expressed in the heart and skeletal muscle. Tenascin-X binds with heparin, fibronectin, laminin, or collagens. Tenascin-X contributes to the stability of ECM of the cardiac tissues (Matsumoto et al., 1994). Rabinovitch and Coll did an *in vitro* experiment. The experiment proposed a functional paradigm of ECM-dependent cell survival where metalloproteinases (MMPs) up-regulate or down-regulate TN-C causing EGF-R clustering and EGF-dependent growth or apoptosis, respectively (Jones and Jones, 2000). *In vitro*, TN-C mRNA and protein accumulation are induced by mechanical stress in neonatal rat cardiomyocytes in an amplitude-dependent manner. Stimulation occurs through a nuclear factor-kappa B-dependent and an angiotensin II-independent mechanism (Yamamoto et al., 1999).

#### 2.9.3 Secreted protein acidic and rich in cysteine

SPARC is a non-structural glycoprotein of the ECM. SPARC was first detected in bones so they are also known as osteonectin (Termine et al., 1981) SPARC shows a high affinity toward collagen and calcium. During infarct healing, SPARC facilitates collagen crosslinking (Schellings et al., 2009). This crosslinking can be detrimental if it is induced by pressure overload and ageing and it can lead to diastolic dysfunction (Komatsubara et al., 2003). SPARC contains properties of the de-adhesion (Murphy-Ullrich, 2001). It interacts and modulates the activity of various growth factors, such as FGF-2, vascular endothelial growth factor, platelet-derived growth factor, insulin-like growth factor-I, and TGF-β which are crucially involved in tissue repair, angiogenesis, and fibrosis (Motamed et al., 2002) (Francki et al., 1999).

#### 2.9.4 Osteopontin

Osteopontin is a matricellular glycoprotein. Osteopontin expression is highly increased during injury (Wang and Denhardt, 2008). The integrin-binding site in osteopontin is activated by the thrombin (Waller et al., 2010). Osteopontin contains a calcium-binding domain. Levels of osteopontin are found elevated in the experimental models of cardiac fibrosis and hypertrophy. The osteopontin expression is also found high in MI, valvular disease, and ageing. Osteopontin increases collagen deposition and reduces chamber dilation post-MI (Trueblood et al., 2001).

#### 2.9.5 Periostin

Periostin is a matricellular glycoprotein. Periostin is involved in the epithelial–mesenchymal transition, healing, and cardiac development. Periostin has elevated expression in the case of myocardial injury. It can interact with integrin. Periostin contributes to cardiac fibrosis; it stimulates fibroblasts and collagen deposition in response to injury (Ladage et al., 2013).

### 2.10 CCN protein family

CCN family consists of six members. It is named CCN because of its three members: cysteine-rich protein 61, connective tissue growth factor, and nephroblastoma-overexpressed protein. Earlier, these were considered as growth factors, but later, studies found them working as cell–extracellular matrix adhesion modulators because of their interactions with proteoglycans, integrins, growth factors, and cytokines (Ladage et al., 2013). The CCN level increases in response to myocardial injury, especially, the connective tissue growth factor (CTGF) increases (Ohnishi et al., 1998). The expression of CTGF is elevated in response to MI and pressure overload. CTGF aids in fibrotic, angiogenic responses, and TGF-β signaling. CCN5 has contrasting effects. CCN5 reduces the fibrotic response and hypertrophy by affecting the TGF-β signaling (Yoon et al., 2010).

### 2.11 Membrane receptors

Cell-surface receptors are very important for the communication of ECM components with cellular components. Cell-surface receptors play a pivotal role in the regulation or progression of cardiac fibrosis and heart failure. Cell-surface receptors such as integrins, discoidin domain receptors (DDR), Fc receptor gamma (FcRy), and toll-like receptors (TLR) are associated with the fibrotic response (Haudek et al., 2008; Borza et al., 2018; Katare et al., 2020; Yokota et al., 2020). Integrins are important for the mechanotransduction (Valencik et al., 2006). Integrin plays a pivotal role in cardiac fibrotic responses (Chen C. et al., 2016). The integrin can sense mechanical stress in the ECM and induce a fibrotic response (Yokota et al., 2020). In addition to integrin, DDR can also induce a fibrotic response. DDR is one of the main targets for combating fibrosis. It is one of the key players involved in the fibrotic response (Borza et al., 2018; Moll et al., 2019). TLR mediates inflammatory pathways. TLR activation has also been found to increase cardiac fibrosis in rats (Katare et al., 2020). Cell-surface receptors can also regulate cardiac fibrosis. It was found that FcRy regulates fibrotic response after binding with serum amyloid *p* (SAP). Deletion of FcRy in mice led to failure in inhibiting the development of cardiac fibrosis and heart failure by SAP administration (Haudek et al., 2008). However, FcRy also mediates the pathway of activation of platelets by collagen. This platelet activation pathway leads to the neointima formation in the left femoral arteries of mice (Konishi et al., 2002).

### 2.12 Next-gen proteomics approaches for better understanding the ECM during cardiac fibrosis

**FIGURE 2:**
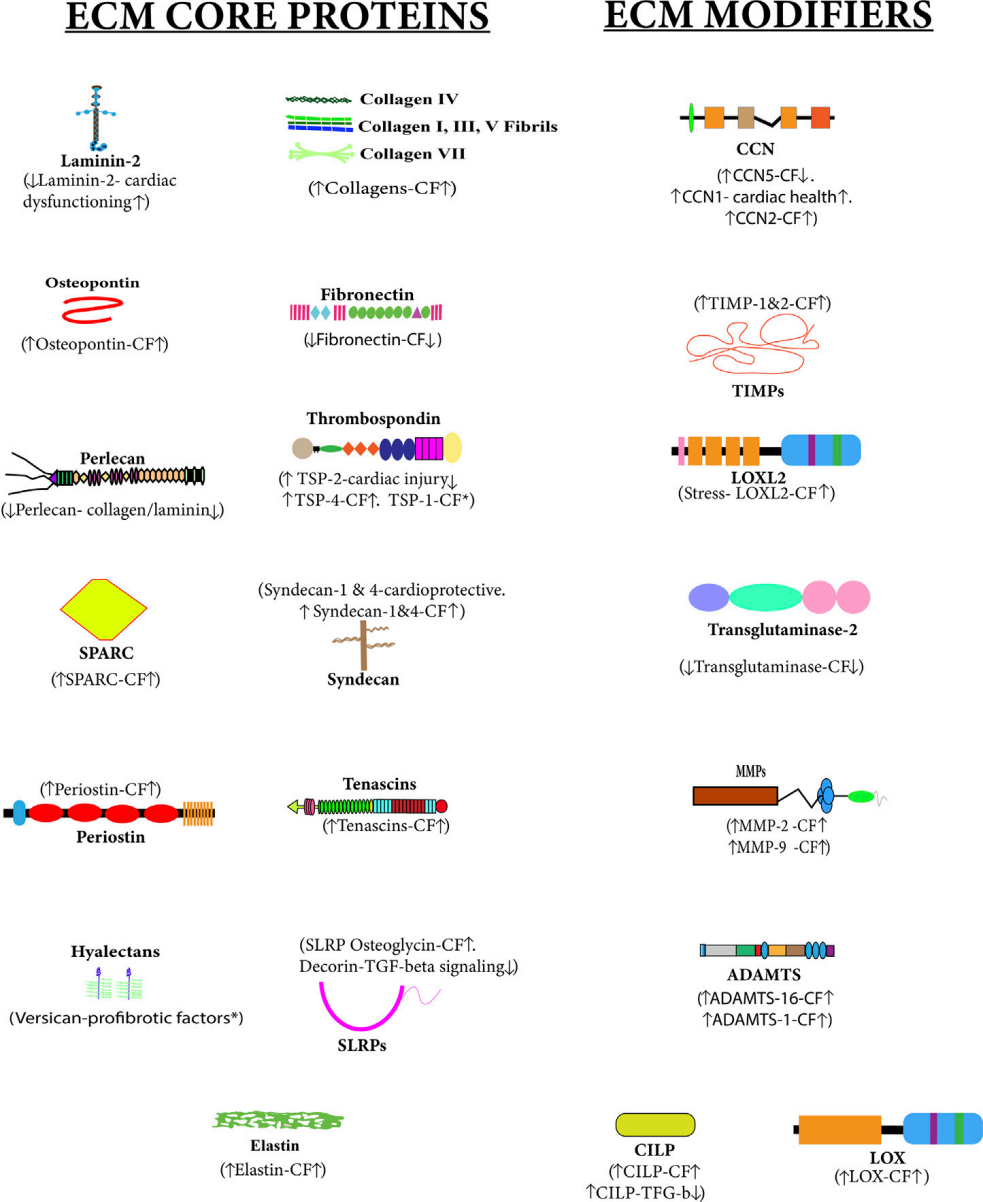
Altered cardiac matrisome during cardiac fibrosis—extracellular matrix (ECM) core proteins and ECM-modifier proteins associated with cardiac fibrosis are shown in this figure. The upside arrow (↑) shows up-regulation, and the downside arrow (↓) shows down-regulation. There is a direct or inverse correlation between the level of some ECM proteins during cardiac fibrosis (CF). Versican and TSP1 have been reported to have pro-fibrotic activities.

Dysregulated ECM remodeling results in the development of cardiac fibrosis. Figure 2 shows the ECM proteins altered during cardiac fibrosis. MS-based proteomics has been utilized in many studies done on cardiac ECM (Suna et al., 2018; Garcia-Puig et al., 2019; Barallobre-Barreiro et al., 2021b). A proteomics and transcriptomics study performed to understand the role of calcitonin on atrial fibrosis showed that the absence of calcitonin increases atrial fibrosis and overexpression of calcitonin prevents atrial fibrosis. One more interesting observation from this study was that the transcriptomic study of human atrial fibroblast showed little change after calcitonin exposure, but the proteomics study covered calcitonin exposure in greater detail and extensive alteration in ECM proteins and pathways related to fibrosis was observed by proteomics (Moreira et al., 2020). This study highlights the significance of proteomics in understanding cardiac fibrosis. Proteomics studies have been significant in deciphering the role of cardiac-ECM remodeling during cardiac fibrosis. The role of ECM remodeling in heart failure, myocardial infarction (MI), atrial fibrogenesis, aortic dilation, post-stent neointima formation, ischemia/reperfusion injury, and zebrafish heart regeneration has been studied with proteomics (Barallobre-Barreiro et al., 2012; 2021b; Fava et al., 2018; Suna et al., 2018; Daseke et al., 2019; Garcia-Puig et al., 2019; Moreira et al., 2020; Chalise et al., 2021). Along with these cardiac ECM protein-level studies, one in-depth site-specific collagen PTM study has also been done on the zebrafish heart (Sarohi et al., 2022b). Although cardiac ECM remodeling has been studied by proteomics in various cardiovascular disease conditions, identification and quantitation of the total ECM proteins have remained a challenge. Human matrisome has 274 core matrisome proteins and 753 matrisome-associated proteins (Nauroy et al., 2018b). However, these 1,027 proteins are not yet identified in any proteomic-based cardiac ECM study. NGP can be utilized to increase the depth of cardiac matrisome coverage with optimized ECM enrichment methods in conjunction with high-resolution MS. Quantitative proteomic analysis of alteration in total ECM proteins and PTMs with NGP will provide deeper insights into the progression of ECM remodeling during cardiac fibrosis. It will also help in identifying key ECM proteins/PTMs specifically altered during cardiac fibrosis, which can further be explored as a biomarker for early diagnosis of cardiac fibrosis.

## 3 Is fibrosis always bad?

Fibrosis is generally associated with a decline in tissue functionality leading to organ failure. However, inherently, it is a fundamental repair response of a failing heart to restore the function of an injured tissue (Diegelmann and Evans, 2004). However, dysregulation in the degree of inflammation and repair may lead to diseased conditions through the development of tissue-scarring (White and Mantovani, 2013). Fibrosis has remained one of the main pathophysiological mechanisms of many CVDs. ECM deposition is a general repair response to any injury/insult or stress in the cardiac tissues (Diegelmann and Evans, 2004). ECM gets deposited in the damaged tissue to prevent further tissue damage and to initiate the reparation/healing process. In the initial phase of the healing process, collagens and other ECM proteins get deposited excessively in the cardiac ECM. Then, regeneration of tissue starts and excessive collagen with other ECM proteins get cleared from the tissue. So, excessive ECM protein deposition plays a significant role in the healing/reparation process of the tissues. This phenomenon has been vividly documented in some fishes and urodeles as they have evolved to master cardiac regeneration. However, dysregulation in the healing/repair process leads to irreversible excessive ECM protein deposition, which results in fibrosis. In fibrosis, ECM proteins are not cleared from the tissue and this excessive deposition causes the formation of scar tissue. Surprisingly, mammals do not have the capability to utilize the fibrotic response as a healing mechanism. On the contrary, Zebrafish can regenerate the injured/amputated heart. It is important to note that in zebrafish, excessive ECM proteins get deposited in the cardiac tissues, but it does not result in pathological fibrosis. As the regeneration proceeds, the excessively deposited ECM proteins are cleared from the cardiac tissues and the heart comes back to its normal state. Recently, our group has shown site-specific PTM changes on different collagen chains during the zebrafish heart-regeneration (Sarohi et al., 2022b). Analyzing the ECM protein repertoire and their post-translational modifications (such as glycosylations, hydroxylations, and crosslinking) may provide key insights into the possibilities of mammalian cardiac regeneration.

## 4 Development of cardiac fibrosis

**FIGURE 3:**
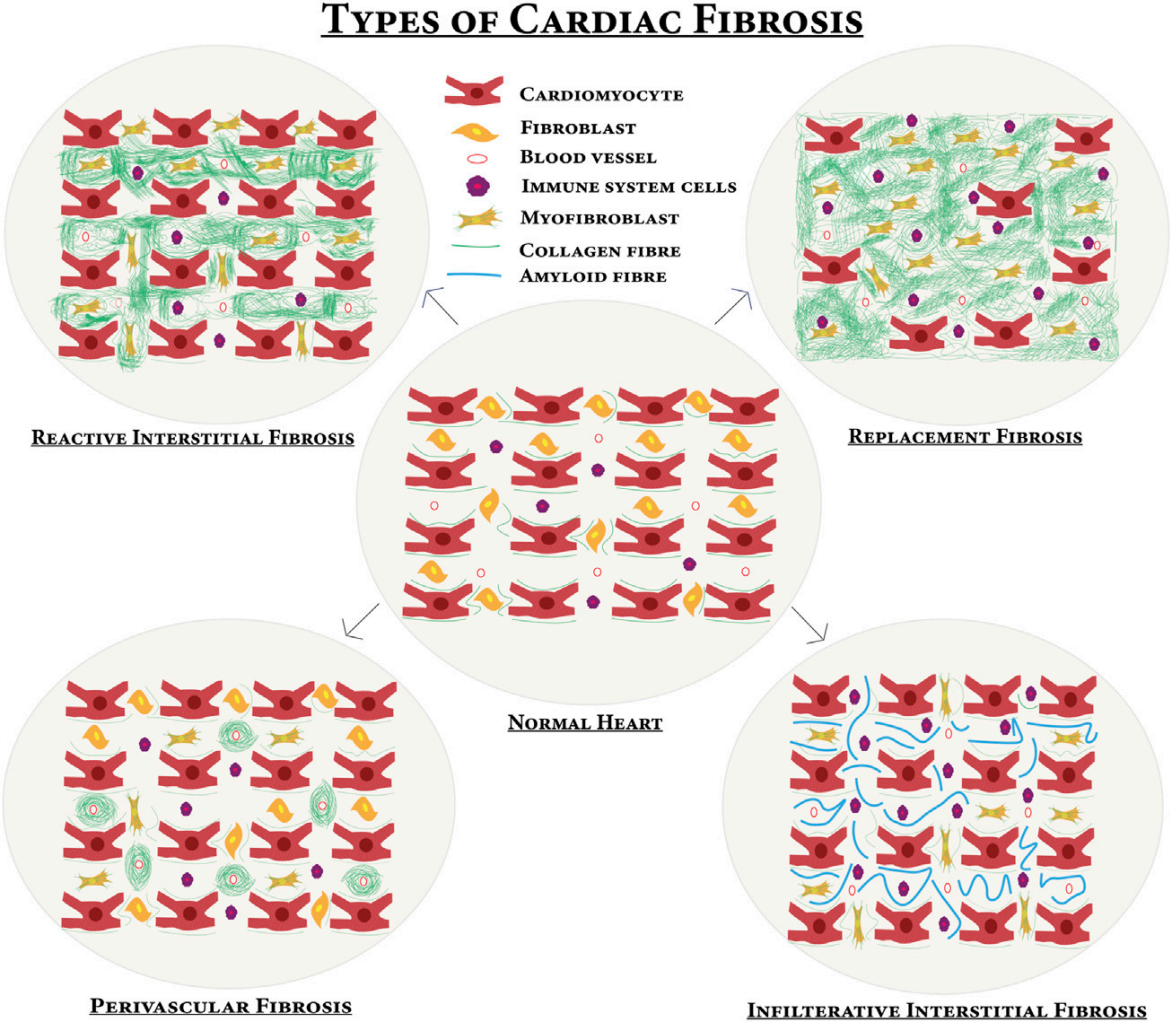
Different types of cardiac fibrosis compared with normal myocardium—upon certain profibrotic stimulations, the normal heart turns into a fibrotic one. In reactive interstitial fibrosis, the collagen is increased but the viability of the myocardium persists. In replacement fibrosis, cardiomyocytes are replaced by fibrosis and cardiomyocytes die, and then extensively produced collagen occupies the space of the dead cardiomyocytes and the myocardium fails to perform the contractile function. Infiltrative interstitial fibrosis is found in amyloidosis or Anderson–Fabry disease and there is inflammatory cell infiltration. A new matrix is produced around blood vessels in perivascular fibrosis.

4.1 Fibrotic response starts with the initiating phase with elevated levels of circulating and myocardial pro-fibrotic growth factors and cytokines (Kong et al., 2014a). In the effective phase, pro-fibrotic growth factors and cytokines bind to their receptors and lead to the activation of specific signaling pathways. This results in the transformation of cardiac fibroblasts (CFs) into myofibroblasts, which expresses the highly contractile protein *a*-SMA and produces different matrix metalloproteinases (MMPs) and tissue inhibitors of metalloproteinases (TIMPs) to regulate the homeostasis of ECM (Kong et al., 2014a; Frieler and Mortensen, 2015). Cardiac fibrosis occurs during ischemic heart disease, MI, hypertrophic cardiomyopathy, diabetic cardiomyopathy, and dilated cardiomyopathy (Hinderer and Schenke-Layland, 2019; Sarohi et al., 2022a). Replacement/reparative fibrosis occurs in MI after cardiomyocyte loss. Cardiac fibrosis can be of four types as shown in Figure 3.

The diagnosis of cardiac fibrosis has remained a challenge in the clinical setting. Biomarkers are usually based on an elevated cardiac collagen deposition that can be a significant feature/sign for many if not every cardiovascular diseases. Pro-collagen types I and III and their propeptides that reflect collagen synthesis (Weber et al., 1994b) and collagen turnover can be investigated by detecting products of matrix metalloproteinase action on collagen in the blood. From the serum collagen peptide concentrations, (i) biomarkers of collagen synthesis: PINP (pro-collagen type I N-terminal propeptide), PICP (procollagen type I C-terminal propeptide), and PIIINP (procollagen type III N-terminal propeptide); and (ii) a biomarker of collagen degradation: ICTP (collagen type I C-terminal telopeptide) (Prockop and Kivirikko, 1995; Querejeta et al., 2004; Fernández-Real et al., 2012; Flevari et al., 2012; López et al., 2012). It is confirmed that PICP is a promising fibrosis marker that is both minimally invasive and reproducible (Klappacher et al., 1995; Martos et al., 2009; Massoullié et al., 2019). The levels of MMP-2 and MMP-9 are also found to be elevated in cardiac fibrosis condition (Medeiros et al., 2019). Not only protein biomarkers but MRI-based techniques (T1, T2 relaxation times, proton density, Gadolinium CMR Enhancement Technique, Gadolinium-Enhanced CMR of Myocardial Fibrosis (MI), and Myocardial T1 mapping) are also quite useful in the detection of the cardiac fibrosis (Judd et al., 1995; Kim et al., 1996; Messroghli et al., 2004; Croisille et al., 2006; Schelbert et al., 2015). However, cardiac fibrosis-specific biomarker has yet not been delineated.

### 4.1 Transdifferentiation of cardiac fibroblast into myofibroblast

The following pathways induce fibroblast transdifferentiation and collagen deposition leading to cardiac fibrosis. Many recent studies have been directed toward understanding the source, development, and activation of fibroblasts into myofibroblasts that can be targeted to develop better anti-fibrotic therapies.

#### 4.1.1 Smad-dependent pathway

**FIGURE 4:**
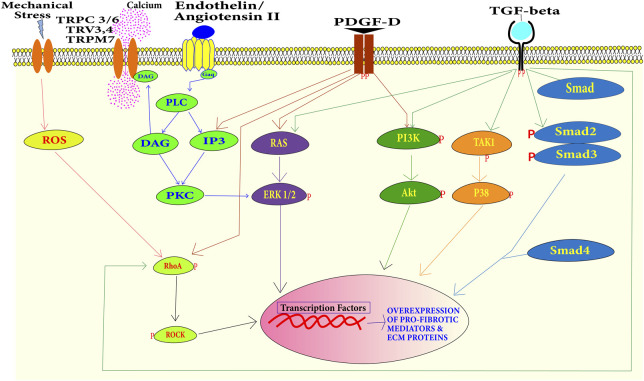
Summary of the signaling pathways during cardiac fibrosis–the binding of profibrotic factors including TGF-β, Ang-II, PDGF, endothelin-1, and mechanical stress has cascading effects. Activation of Smads leads to the transcription of excessive ECM proteins and pro-fibrotic mediator proteins. Transcription of these myofibroblast phenotypic factors can also be activated by PI3K/Akt, TAK1/P38 MAPK, RAS/Erk MAPK, and RhoA/ROCK pathways.

The Smad-dependent pathway operates in response to TGF-β. TGF-β binds with the TGF-β type II receptor and then the TGF-β type II receptor activates the TGF-β type I receptor. The activated TGF-β type I receptor activates Smad2 and Smad3. Smad4 forms a heterotrimeric complex with Smad2 and Smad3 (Figure 4). This heterotrimeric complex translocates into the nucleus and binds with Smad-binding elements (SBE). It recruits the CREB-binding protein or p300 to enhance the transcription of profibrotic genes. Smad3 null mice study confirmed the role of Smad signaling in cardiac fibrosis (Bujak et al., 2007). Smad6 and Smad7 have an inhibitory effect on TGF-β type I receptor, they can even induce the degradation of TGF-β type I receptor. Smads also modulate the expression of microRNAs. Modulation of microRNA expression by Smads is on both the post-translational level and the transcriptional level (Blahna and Hata, 2012).

#### 4.1.2 Smad-independent pathways

Cardiac fibrosis can also be developed without the involvement of Smads (Figure 4). TGF-β can induce signaling independent of Smads as well. A study reported that MAPK can be activated in response to TGF-β in Smad null cells. This indicates that MAPK activation is independent of Smad signaling (Engel et al., 1999). TGF-β binding to TGF-β type II receptor activates it. This activated receptor activates TGF-β-activated kinase 1 (TAK1). TAK1 activation leads to the activation of p38 (Wang et al., 1997). Extracellular-regulated protein kinases (ERK) can also get activated independently of Smad through small GTPase Ras under growth factor stimulation. In a study on Marfan mice ERK, it was found to induce cardiac fibrosis (Rouf et al., 2017). MAPK and ERK can also activate the Smad-signaling pathway. ERK can up-regulate the TGF-β expression and p38 can activate a Smad interacting transcriptional factor Activating Transcriptional Factor-2 (ATF2) (Massagué, 2000). Phosphoinositide-3 kinase (PI3K) can also be activated through the growth factor receptor. Activated PI3K can activate the Protein Kinase B, well-known as Akt. Activated Akt induces mTOR downstream signaling. This signaling eventually leads to the transcription of profibrotic genes and the development of cardiac fibrosis and excessive scarring (Aoyagi and Matsui, 2011). Activated RhoA activates the Rho-associated coiled–coil-containing kinases (ROCKs). RhoA can be activated by a growth factor stimulus or by reactive oxygen species. ROCKs are downstream mediators of the effects of activated RhoA. Along with regulating the actin cytoskeleton, ROCK phosphorylate targets are important for remodeling including myosin light chain (MLC) and myosin-binding subunit (MBS) on MLC phosphatase (MLCP). Up-regulation of ROCK leads to cardiac remodeling (Shimizu and Liao, 2016). The Wingless/int1 (WNT) signaling pathway also plays a role in the development, proliferation, and differentiation of cardiac cells (Foulquier et al., 2018).

#### 4.1.3 Smad linker phosphorylation

Phosphorylation of the linker region of Smads modulates the Smad-dependent TGF-β pathway. Smads have three regions—MH1, linker, and MH2 (Burch et al., 2011). Canonical Smad-dependent TGF-β signaling works through the phosphorylation of the MH2 region (C-terminal) of Smads (Euler-Taimor and Heger, 2006). However, the linker region of Smads can also be phosphorylated by kinases activated by both Smad-dependent and -independent pathways. Activated p38 MAPK, ERK, Jnk, TAK1, ROCK, calcium-calmodulin dependent (CAM) kinase, glycogen synthase kinase-3 (GSK3), and cyclin-dependent kinase (CDK) can phosphorylate the linker region of Smads (Burch et al., 2011; Kamato et al., 2020). Initially, it was found that phosphorylation of the linker region prevents the translocation of Smads into the nucleus and inhibits the Smad-dependent TGF-β signaling pathways. But later, it was found that Smads phosphorylated at the linker region which can be translocated into the nucleus and stimulate gene expression (Kamato et al., 2020). A study performed on human mesenchymal stem cells showed that interleukin-1β (IL-1β) delays the TGF-β signaling by Smad linker phosphorylation mediated by TAK1 (van den Akker et al., 2017). Smad linker phosphorylation also contributes to cardiac pathophysiologies. Smad2 linker phosphorylation can stimulate the synthesis of biglycan and hyperelongation of the GAG chains (Burch et al., 2010; Rostam et al., 2016). Hyperelongated GAG chains facilitate the binding of lipoproteins onto the walls of blood vessels (Rostam et al., 2016). Lipoprotein accumulation on the blood-vessel walls results in atherosclerosis leading to heart failure (Rostam et al., 2016). Phosphorylation on the linker and C-terminal region of the Smads mediate different cellular functions. Region-specific phosphorylation of Smads exerts dynamic complexity in gene expression. Phosphorylation of Smads can be potentially targeted for the management of cardiovascular diseases.

### 4.3 NGP approaches for understanding the development of cardiac fibrosis

Transdifferentiation of cardiac fibroblast into myofibroblast upon stimulation through various profibrotic factors is a key process in the development of cardiac fibrosis. Both Smad-dependent and Smad-independent pathways involve the cascading downstream processes. The major proteins of these pathways are regulated by the process of phosphorylation (Figure 4). These pathways are well-studied in fibroblast transdifferentiation. However, the immunoblotting technique is widely used to detect the activation of various components of these pathways by detecting the phosphorylated epitope of a particular protein in a signaling cascade. Although immunoblot is a reliable and gold-standard technique, only a limited number of candidates (only those proteins with specific phosphorylation antibody availability) involved in a pathway can be evaluated. In a fibroblast that is transdifferentiating into myofibroblast, there can be different profibrotic pathways active in real-time that are cumulatively contributing to the transdifferentiation process. A more global approach is required to map these real-time complex signaling cascades. Dissecting and mapping the dynamic kinome across all the samples at the same time requires more rigorous, specific MS-based approaches to biologically and technically relevant data. These challenges can only be overcome by utilizing MS-based proteomics (Humphrey et al., 2018; Savage and Zhang, 2020; Liu et al., 2021). The field of phosphoproteomics has emerged greatly in the last decade (Lundby et al., 2013; Sharma et al., 2014; Alam et al., 2015; Riley and Coon, 2016; Steger et al., 2016; Tan et al., 2017; Kuzmanov et al., 2020a; Wu et al., 2020; Sims et al., 2022). It has contributed significantly to biological research. Lundby et al. studied the β-adrenergic receptor signaling in murine heart using phosphoproteomics (Lundby et al., 2013). β-Adrenergic receptors signaling increases the heart rate by increasing cardiomyocyte contractility. They treated mice with β-adrenergic receptors agonist and antagonist. They found that activation of the potassium ion channel by phosphorylation was associated with increased heart rate (Lundby et al., 2013). Kuzmanov et al. screened for potential targets to reduce cardiac fibrosis utilizing phosphoproteomics in hypertrophic cardiomyopathy patients and mice models of cardiac fibrosis. They found GSK-3 to be a consistent mediator of fibrosis and the inhibition of GSK-3 resulted in reduction of cardiac fibrosis (Kuzmanov et al., 2020b). Optimized phosphorylation enrichment followed by NGP can provide finer molecular level signaling details about the total kinome (phosphoproteome) of the transdifferentiating myofibroblast. Analysis of total phosphoproteome can decipher the phosphorylated components of the different active pathways. This can also be used in studying the synergistic and antagonistic effects of different pathways. Analysis of phosphoproteome can also help in the identification of phosphoproteins highly contributing to the development of cardiac fibrosis. These phosphoproteins can be targeted to regulate cardiac fibrosis.

## 5 Major contributors of cardiac fibrosis

**FIGURE 5:**
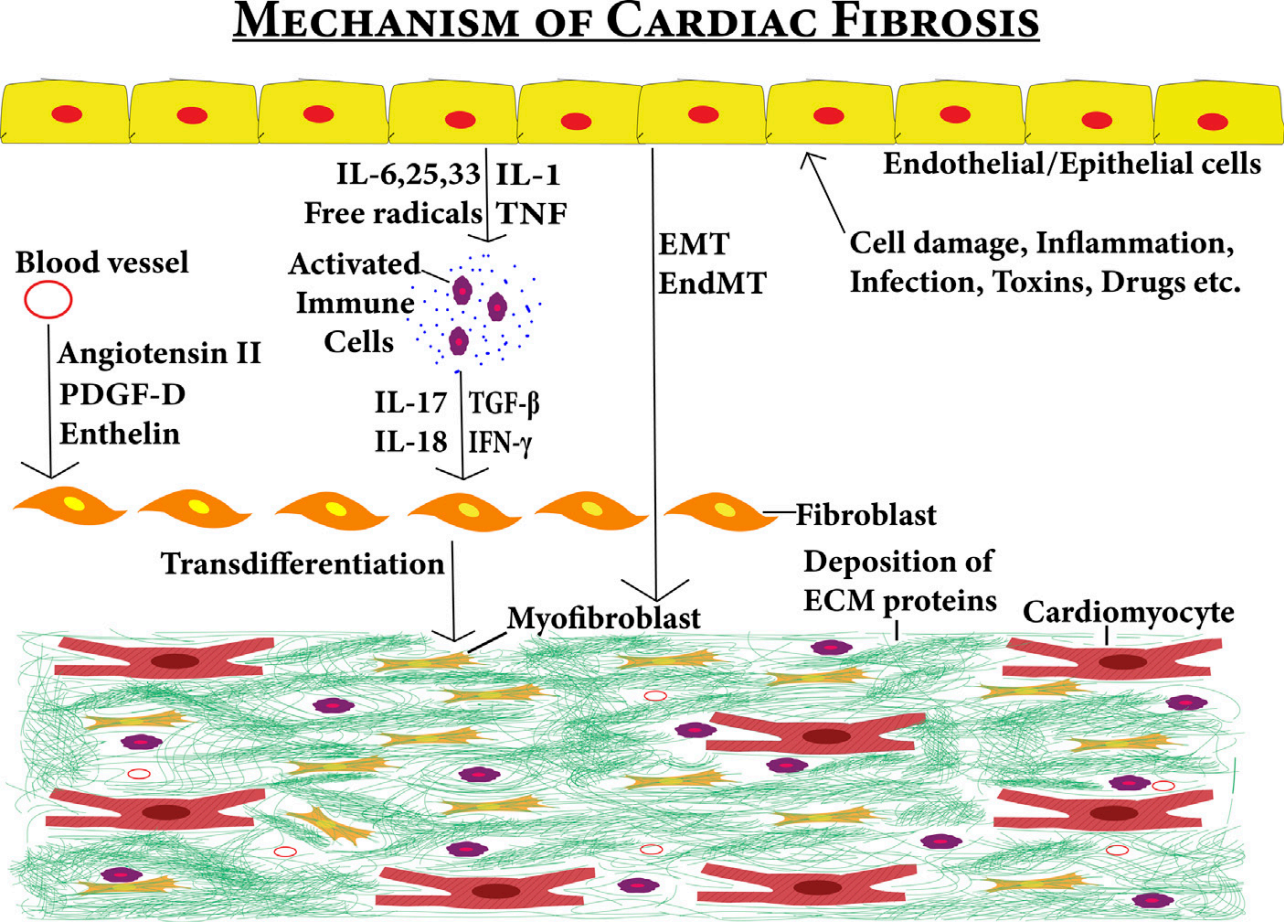
Fibrotic niche contributed by several stimuli–the figure shows the various factors contributing to the development of cardiac fibrosis. Effects of various stimuli (cell damage, inflammation, infections toxins, and drugs) on epithelial/endothelial cells can induce cardiac fibrosis either directly *via* EMT/End MT pathways or indirectly by first activating immune system cells *via* cytokines. Then, the activated immune system cells secrete profibrotic cytokines leading to the transdifferentiation of cardiac fibroblasts into myofibroblasts. The circulation system also contributes to cardiac fibrosis by entering the profibrotic factors into the cardiac tissues. These profibrotic factors also induce cardiac fibroblasts to myofibroblast transdifferentiation leading to cardiac fibrosis.

The development of cardiac fibrosis is a complex process. Cellular damage, inflammation, drugs, toxins, oxidative stress, and mechanical stimulus such as pressure overload may trigger transdifferentiation of fibroblasts into specialized cells termed as myofibroblasts. The transdifferentiation of fibroblast to myofibroblast can be formed due to direct stimulants from the inflammatory cells and circulatory stimulants from the blood vessels (Lee et al., 1995; Testa et al., 1996; Campbell and Katwa, 1997; Bujak et al., 2007). But, transdifferentiation can also be stimulated from endothelial, epithelial, and cardiomyocyte cells (Hay, 1995; Thiery, 2002; Zeisberg et al., 2007). These cells under certain conditions secrete profibrotic agents that either stimulate fibroblast directly or first stimulate immune system cells which then stimulate fibroblast to proliferate into myofibroblast. These triggers can be in the form of signaling molecules such as TGF-β1, fibroblast growth factor (FGF), endothelin-1, and cytokines such as IL-1, IL-6, and TNF-α (Schroer and Merryman, 2015). The myofibroblast cells not only arise from fibroblasts (resident or bone-marrow source) but can also arise from epithelial-to-mesenchymal transitions (EMT) and endothelial-to-mesenchymal transitions (EndMT) (Kong et al., 2014b). These contributors can be targeted to combat cardiac fibrosis (Figure 5).

### 5.1 ECM processing and deposition

Collagens I and III are majorly synthesized by myofibroblasts which are then processed to attain their mature form due to the action of lysyl oxidase. Mature collagen chains cross-link together and form a mesh of fiber. Collagen I which contributes to rigidity is observed to be deposited in higher concentrations. This leads to increased tension in the ventricular wall (Segura et al., 2014). Stimulating factors from myofibroblasts promote further transdifferentiation of fibroblasts and lead to more ECM remodeling (Bonnans et al., 2014). Other factors such as ageing and metabolic dysfunctional conditions such as those seen in diabetes can also contribute to the exacerbated response of collagen deposition (Prabhu and Frangogiannis, 2016).

### 5.2 Immune and inflammatory cell participation

In normal conditions, basal levels of progenitor cells are maintained which gets differentiated into a specialized form under stressed conditions. Examples of such cells are macrophages that are involved in ECM remodeling and those that differentiate from monocytes. During cardiac remodeling, two subsets of macrophages differentiated by heterogeneous markers can be observed (Frangogiannis, 2019). M1 and M2 belong to the macrophage subpopulation associated with a distinct role in remodeling. M1 macrophages have pro-inflammatory action while M2 macrophages have anti-inflammatory and cardioprotective action. The polarization of M1 macrophages from a monocyte is mediated by cytokines TNF-α and interferon-gamma (IFN-γ) secreted from the type 1 T-helper (Th1) cells (Frangogiannis, 2019; Viola et al., 2019; Kim et al., 2021). The bacterial lipopolysaccharide (LPS) is also a potent mediator of M1 polarization (Kim et al., 2021). The pro-inflammatory cytokines TNF-α, IL-1α, IL-1β, IL-6, IL-12, and IL-23 are secreted by M1 macrophages (Voll et al., 1997; Kim et al., 2021). M1 macrophages are discerned to be engaged in proteolytic and signaling activities (secretion of TNF, IL-1, or ROS) throughout the inflammatory stage, specifically in the infarcted area. M1 macrophages mediate the removal of dead cells and ECM debris. Cytokines secreted from M1 macrophages gradually increase the infarct size (Duncan et al., 2020; Chen et al., 2022). Contrastingly, M2 macrophages are involved in drafting fibroblasts to the injury site and secreting pro-fibrotic mediators such as IL-10, TGF-β, and platelet-derived growth factor (PDGF) (Frangogiannis, 2019; Viola et al., 2019; Kim et al., 2021). The M2 macrophages initiate an anti-inflammatory response to drive the healing processes. M2 macrophage polarisation from the monocyte is mediated by IL-4 and IL-13 secreted by Th2 cells (Voll et al., 1997; Viola et al., 2019). A study performed on a mice model of doxorubicin-induced heart failure showed that administration of M2-like macrophages improved heart function and reduced cardiomyocyte apoptosis (Liu et al., 2022). Shiraishi et al. showed that the population of M2-like macrophages was increased in infarcted areas in mice hearts (Shiraishi et al., 2016). The selective depletion of M2-like macrophages in mice resulted in frequent cardiac rupture, reduced collagen fibril formation in infarcted areas, and decreased tissue repair post-MI (Shiraishi et al., 2016). MMP-28 facilitates M2 macrophage activation. The deletion of MMP-28 in mice resulted in MI-induced ventricular dysfunction and rupture. Collagen synthesis was also reduced in MMP-28 mice post-MI (Ma et al., 2013). These studies indicate that M2 macrophages are required to facilitate the healing process in cardiac tissues. Certain pro-fibrotic and inflammatory functions are also mediated by mast cells (Levick Goldspink, 2014). The contents secreted by mast cells such as histamine can act as proliferative agents for fibroblasts and stimulate their activation during pulmonary fibrosis (Veerappan et al., 2013). It also participates in the process of cardiac remodeling (Levick Goldspink, 2014). T-cells are well-known to be associated with heart failure progression. Nevers et al. showed that heart failure samples from transverse aortic constriction (TAC) mice and non-ischemic patients had increased affinity and higher adhesion for activated vascular endothelium. Furthermore, T-cell recruitment can promote pathological cardiac remodeling during heart failure (Nevers et al., 2015, 2017). Immune cells and cytokines secreted by the immune cells majorly contribute to ECM remodeling. Cytokines leading to dysregulated ECM remodeling can be therapeutically targeted and cardioprotective cytokines can be further explored for better management of cardiovascular diseases.

### 5.3 Epithelial-to-mesenchymal transition and endothelial-to-mesenchymal transition

EMT is a type of cellular switching proven to be very important in biological programs. The expression of epithelial markers is lost during this process of losing polarity and adhesion properties (Hay, 1995; Thiery, 2002). Meanwhile, the expressions of mesenchymal markers such as smooth muscle *a*-actin (SMA) and fibroblast-specific proteins emerge (Zeisberg et al., 2007). Additionally, phenotypes corresponding to migration and transdifferentiation are attained. Zeisberg *et al*. explored that one of the origins of the fibroblast is the transition from endothelial cells by performing the lineage analysis in the murine model of cardiac fibrosis which is extended to prove that it promotes the progression of the same. Although it is shown that TGF-β in the same model triggers EndMT through Smad3 transcriptional activity, on the other hand, the recombinant form of human BMP-7 i. e rhBMP-7 reduces EndMT (Zeisberg et al., 2007)**.** It was found that under a diabetic milieu**,** endothelin-1 production increased by vascular endothelial cells contributing to exacerbated cardiac fibrosis through EndMT (Widyantoro et al., 2010). Furthermore, an elevated level of endothelin-1 has been found to be associated with cardiac fibrosis progression among diabetic patients (Takahashi et al., 1990; Widyantoro et al., 2010; Sen et al., 2012; Russo and Frangogiannis, 2016). EMT and EndMT are the processes that accelerate the progression of fibrosis. Fibrosis progression is reduced by inhibiting these processes (Widyantoro et al., 2010). These processes can be therapeutically targeted for inhibition of the progression of cardiac fibrosis.

### 5.4 Blood vessel: An important ECM content source

Angiotensin II is a major protein hormone that acts on the central nervous system causing blood vessels to constrict. This aids in maintaining fluid balance and blood pressure in the body (Lee et al., 1995; Campbell and Katwa, 1997)**.** In the case of cardiac fibrosis, angiotensin II is seen to up-regulate TGF-β1 in fibroblast and myofibroblast *in vivo and in vitro* conditions which consequently affect collagen production (Kupfahl et al., 2000).

### 5.5 Inflammatory cytokines

Inflammatory factors can be released by various cardiac cells. The presence of inflammatory factors correlates with cardiac dysfunction. Elevated levels of IL-1, TNF-α, and IL-6 indicate the severity of cardiac diseases in heart patients (Testa et al., 1996). Overexpression of TNF-α in the heart results in cardiac fibrosis, eventually leads to dilated cardiomyopathy (Kuhota et al., 1997). IL-25,33 activates inflammatory cells. IL-1 and IL-6 demonstrate pleiotropic roles in fibrosis; these cytokines contribute to fibroblast transdifferentiation. IL-6 is found to aid in Ang-II-induced cardiac fibrosis (Ma et al., 2012). The role of IFN-γ in cardiac fibrosis is unclear. It can have a pathological or protective role in cardiac fibrosis (Levick and Goldspink, 2014). IL-17 inhibits MKP-1 (MAPK phosphatase-1) and activates p38 MAPK and ERK 1/2, thus it contributes to cardiac fibroblast transdifferentiation and migration (Valente et al., 2012). Overexpression of IL-18 is found to aggravate the remodeling and fibrosis in the heart (Xing et al., 2010).

### 5.6 NGP approaches for identifying potential targets for therapeutics of cardiac fibrosis

The aforementioned factors are majorly known for contributing to the development and progression of cardiac fibrosis. Not just the matrisome and phosphoproteome, but cardiac tissue-based total proteome studies by NGP can also greatly help in assessing the major contributors of fibrosis and even identify unreported contributors. NGP analysis of total tissue lysate can provide deeper insights into the alteration in cellular and extracellular proteins/PTMs during cardiac fibrosis. These altered proteins/PTMs can be further screened for their specificity during cardiac fibrosis. The role of cardiac fibrosis-specific protein and site-specific PTMs can be determined during cardiac fibrosis. These proteins/PTMs can then be explored as potential targets for the treatment/reversal of cardiac fibrosis which is not curable as of now.

## 6 Available treatment of cardiac fibrosis

Pharmaceutical agents are generally used in the treatment and control of cardiac fibrosis. Mechanical devices are used to support heart functions in patients with severe cardiac fibrosis or during high-dose treatment with pharmaceutical agents.

### 6.1 Angiotensin-converting enzyme inhibitors

Experimentally, angiotensin-convertase enzyme inhibitors are found to block cardiac remodeling and reverse the remodeling of cardiac tissues (Klotz et al., 2007). Ang-II is generated by proteolytic cleavage of Ang-I by ACE. Ang-II is a profibrotic agent (Kawano et al., 2000; Díez, 2004). Because ACE inhibitors block the Ang-II formation, they are used in the treatment of cardiac fibrosis.

### 6.2 Angiotensin receptor blockers

Angiotensin receptor blockers prevent vasoconstriction and are used for the treatment of cardiac fibrosis. Prolonged treatment with ARB can reverse the remodeling in a patient having cardiac fibrosis (Granger et al., 2003). ARBs can be prescribed to patients who have ACE inhibitor intolerance.

### 6.3 Beta-adrenergic receptor (β) blockers

High levels of catecholamines significantly contribute to the progression of cardiac diseases. Beta-adrenergic receptor (β) blockers temporarily reduce the blood pressure and progression of heart diseases. β-blockers are helpful in improving the systolic function and reducing the dimensions of the left ventricle. Studies on β-blockers reveal that they increase the survival rate of heart patients (Domanski et al., 2003).

### 6.4 Aldosterone antagonists

A high expression of aldosterone in the body causes mineral imbalance. Aldosterone increases the level of sodium in the body and decreases water and potassium levels in the body. Aldosterone receptors are highly expressed in cases of heart failure. Treatment with aldosterone receptors improves cardiac function and reduces remodeling in patients having cardiac fibrosis (Pitt et al., 1999).

### 6.5 Mechanical assistive devices

#### 6.5.1 Left ventricle-assist devices

Mechanical devices are used to assist in the cardiac function. LVADs reduce the progression of cardiac diseases. Prolonged use of LVAD with pharmaceutical agents promotes reverse remodeling (Ambardekar and Buttrick, 2011).

#### 6.5.2 Cardiac resynchronization therapy

The biventricular pacemaker device is used in CRT. CRT improves cardiac function, reduces dilation of the ventricle, and promotes reverse remodeling (St. John Sutton et al., 2009).

### 6.6 Novel and emerging therapies

Phosphodiesterase type 5 (PDE5) inhibitors are also used in the treatment of cardiac fibrosis to aid other therapies (Guazzi et al., 2011). PDE5 inhibitors improve the diastolic function. Some microRNAs have cardioprotective natures, these can be utilized for the control and treatment of cardiac fibrosis (Kukreja et al., 2011). Table 1 summarizes the potential drugs for cardiac fibrosis under different phases of clinical trials in the United States (2020–2021).

**TABLE 1:** Drugs and biological agents undergoing trials in the United States in 2020–2021 (ClinicalTrials.gov, 2021).

Drug	Title of project	Description	Disease or condition	Location
Dapagliflozin	Effects of SGLT-2 Inhibition on Myocardial Fibrosis and Inflammation as Assessed by Cardiac MRI in Patients with DM2	This study is conducted to assess the potential of dapagliflozin in SGLT-2 inhibition in patients with myocardial fibrosis and type-2 diabetes	Type 2 Diabetes Mellitus	University of Washington
			Myocardial Fibrosis	Seattle, Washington, United States
			al Inflammation	
Sildenafil	Sildenafil Exercise: Role of PDE5 Inhibition	This study has been conducted to assess the effects of sildenafil (PDE5 inhibitor). Effects include reduction in inflammation and improvement in cardiac functions in patients with cystic fibrosis	Cystic Fibrosis	National Jewish Health
				Denver, Colorado, United States
				Augusta University
				Augusta, Georgia, United States
BYDUREON	Extended Release Exenatide *Versus* Placebo in Diabetic Patients with Type 4 Cardiorenal Syndrome	This study will assess the effects of 38 weeks of bydureon treatment on patients having type 2 diabetes mellitus with cardiorenal syndrome	Type 2 Diabetes Mellitus	Baylor Scott and White Research Institute- Baylor Heart and Vascular Hospital
			Chronic Kidney Disease	Dallas, Texas, United States
			Cardiorenal Syndrome	
Allogeneic Derived Cells	Regression of Fibrosis & Reversal of Diastolic Dysfunction in HFPEF Patients Treated with Allogeneic CDCs		Congestive Heart Failure	Medical University of South Carolina
			Heart Failure, Diastolic	Charleston, South Carolina, United States

## 7 Conclusion

**FIGURE 6:**
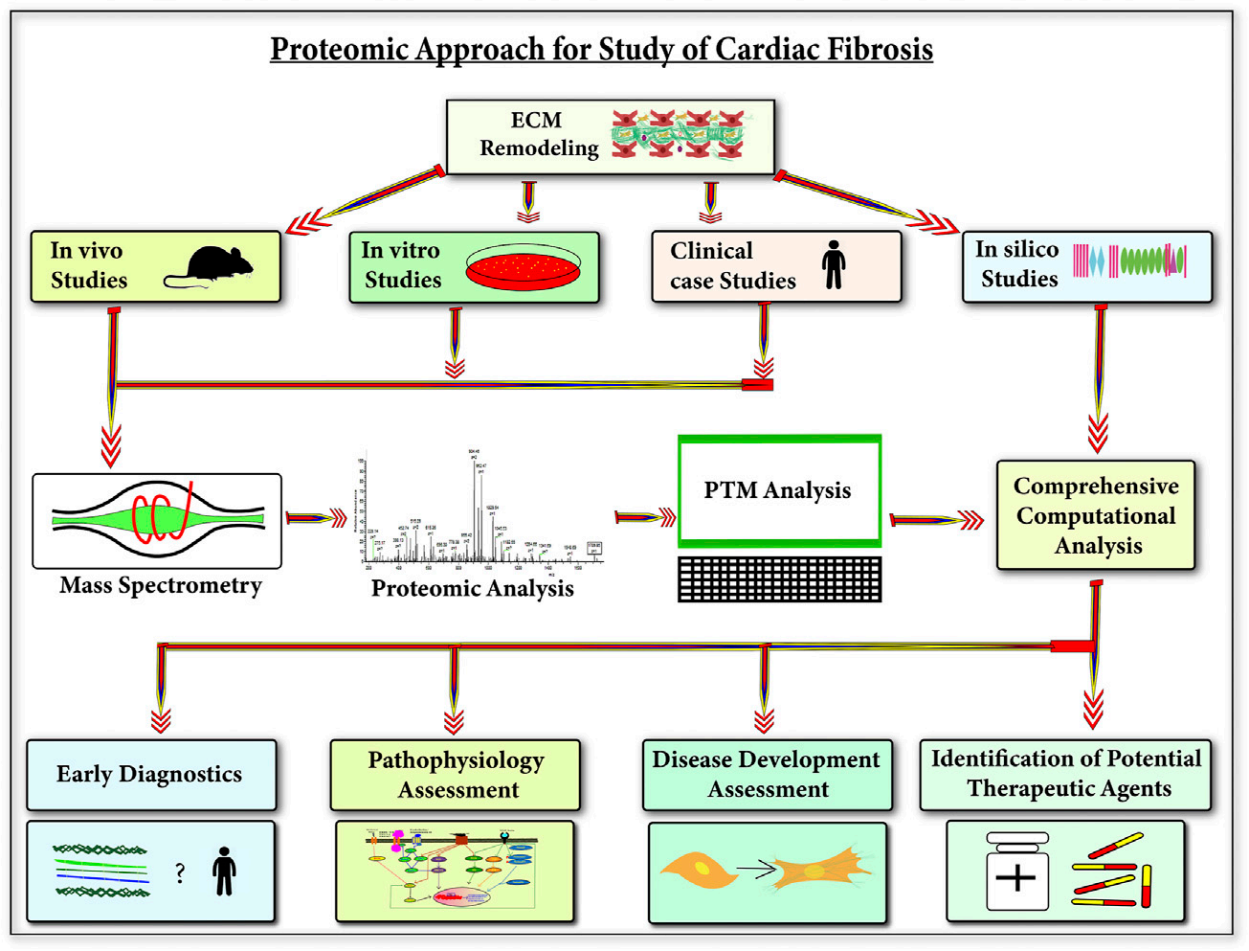
Next-gen proteomics applications to understand cardiac fibrosis—this figure shows the applications of understanding ECM during cardiac diseases using next-gen proteomics. Utilizing next-gen proteomics (protein and PTM analysis) on three biological study types (animal models, cell line models, and clinical human cases) with *in silico* studies can provide an in-depth understanding of the role of cardiac ECM in the development of cardiac diseases. This understanding will be significantly beneficial in the early diagnosis of cardiac diseases, pathophysiology, and development assessment of cardiac diseases and also in the identification of potential therapeutic agents for cardiac diseases.

Dissecting the ECM proteome during cardiac fibrosis is highly important to delineate the complex process of ECM remodeling-mediated scar-tissue formation. Exploring the ECM protein repertoire with mass-spectrometry has remained a challenge mostly because of its insoluble nature. In the recent past, sample-prep methods have been optimized for enriching ECM samples (Lindsey et al., 2018; Barallobre-Barreiro et al., 2021a; McCabe et al., 2021, 2022). Simultaneously, enormous advances (in resolution and sensitivity) in next-gen mass-spectrometry capabilities have increased the depth of analysis. For the last few decades, immunohistochemistry-based staining of collagens (mostly fibrillar) has remained as the gold standard for diagnosing tissue fibrosis. However, proteomics holds the key to discovering new potential biomarkers for cardiac fibrosis patients. Following the success of MudPiT technology in proteomics, recently advanced 4D proteomics have been developed (Yang et al., 2022). Retention time, mass-to-charge ratio, and intensity of the peaks are the three dimensions of MS analysis in ion chromatogram, now the fourth dimension of ion mobility spectrometry (IMS) has been introduced into proteomics (Meier et al., 2021; Yang et al., 2022). In 4D proteomics, peptides are firstl separated in nanoLC and they are again separated in the gas phase in IMS. These two different types of separations provide a higher resolution resulting in higher sensitivity in the identification of peptides (Meier et al., 2021; Yao et al., 2021; Yang et al., 2022). Co-eluting and fusion peptides can also be accurately analyzed by IMS-MS. The in-depth analysis in proteomics is also greatly affected by the data acquisition mode. Conventionally, dynamic exclusion in the data-dependent acquisition (DDA) limits the identification of low-abundant proteins and site-specific PTMs. In several scenarios, the identification of PSMs (peptide–spectrum matches) at the MS2 level becomes a huge challenge. This leads to the difficulty in the accurate quantitation of low-abundant and co-eluting peptides and peptides with similar mass-to-charge ratios. Recent developments with data-independent acquisition (DIA) are a better approach for the quantitation of proteins and PTMs. In DIA, a targeted extraction of the MS2 ions aligned with its precursor improves the sensitivity and specificity of quantitation. Combining the resolution of IMS-MS with DIA is the next Frontier in the proteomics (Soni, 2022) approach to substantially dig deeper with greater sensitivity and specificity to expand the myocardium-specific ECM protein *repertoire* along with site-specific post-translational modifications. Furthermore, the application of NGP-based ECM proteomics in *in vitro* and *in vivo* models of cardiac fibrosis will not only broaden the mechanistic understanding but may yield potential candidates to tinker with ECM remodeling to combat cardiac fibrosis (Figure 6)**.**
